# Brief biology and pathophysiology of Tekt bundles

**DOI:** 10.1080/19336918.2025.2465421

**Published:** 2025-02-13

**Authors:** Jun Yin, Min Liu, Xiao Wang, Hongming Miao, Wenjuan He, Wei Liu, Zhongying Yu, Qinghua Zhang, Jialian Bai, Yimei Cheng, Bing Ni

**Affiliations:** a Department of Pathophysiology, College of High Altitude Military Medicine, Army Medical University, Chongqing, China; b Department of Infectious Diseases, Chongqing Public Health Medical Center, Chongqing, China; c Department of Immunology, Army Medical University, Chongqing, China; d Department of Urology, The 909th Hospital, School of Medicine, Xiamen University, Zhangzhou, China; e Reproductive Medical Center, Daping Hospital, Army Medical University, Chongqing, China; f School of Artificial Intelligence and Big Data, Chongqing Industry Polytechnic College, Chongqing, China; g Department of Pharmacy, Southwest Hospital, Army Medical University, Chongqing, China

**Keywords:** Cilia, flagella, microtubule inner proteins, Tekt bundle, Ciliopathies

## Abstract

Tektins, a family of microtubule-stabilizing proteins, are critical for cilia and flagella assembly in mammals. They maintain doublet microtubule stability and ciliary/flagellar motility. Loss of Tekt1–5 causes microtubule instability, impaired motility, and diseases like infertility, retinal degeneration, Mainzer-Saldino syndrome, and diabetic nephropathy. Pathophysiological stimuli regulate Tektin expression through transcriptional, posttranscriptional, translational, and posttranslational modifications. This review summarizes the latest findings on Tektin functions and their role in diseases.

## Introduction

Cilia, which are microtubule-based structures in eukaryotic cells, serve dual roles as both sensory antennae and motility propellers. They can be categorized into two types: nonmotile and motile. Nonmotile cilia, often referred to as primary cilia, are characterized by their microtubule arrangements, typically 9x2+2 or 9x2+0, and play a crucial role in signal transduction through pathways such as hedgehog, Wnt, and calcium signaling. On the other hand, motile cilia are responsible for the movement of cells and the propulsion of fluids. Given that cilia are present in cells across various tissues, their malfunction can result in a spectrum of human diseases collectively known as ciliopathies. These conditions encompass a range of disorders, including chronic rhinosinusitis, otitis media, retinal degeneration, asthenospermia, bronchiectasis, and hydrocephalus [1].

The architecture and protein makeup of motile cilia are remarkably consistent across eukaryotic organisms. The axoneme, which forms the central component of motile cilia, is composed of nine pairs of DMTs, a configuration commonly known as the 9×2 arrangement. Motile cilia can be distinguished into two subtypes: those with a central microtubule pair, exhibiting a 9×2+2 structure, such as those found in sperm flagella, respiratory cilia, and ependymal cilia; and those lacking a central pair, characterized by a 9×2+0 structure, like nodal cilia [2]. Within each Doublet microtubule (DMT), 10 protofilaments of the B-tubule interface with 13 protofilaments of the A-tubule at the inner and outer junctions. Key motility structures in motile cilia, including axonemal dyneins, radial spokes, and the nexin–dynein regulatory complex (N-DRC), are positioned along the DMTs with a periodicity of 96 nanometers. Dyneins attached to the A-tubules facilitate sliding against adjacent B-tubules, and this sliding motion is transmitted along the length of the axoneme, creating a bending force that drives ciliary motion.

**Figure 1.**
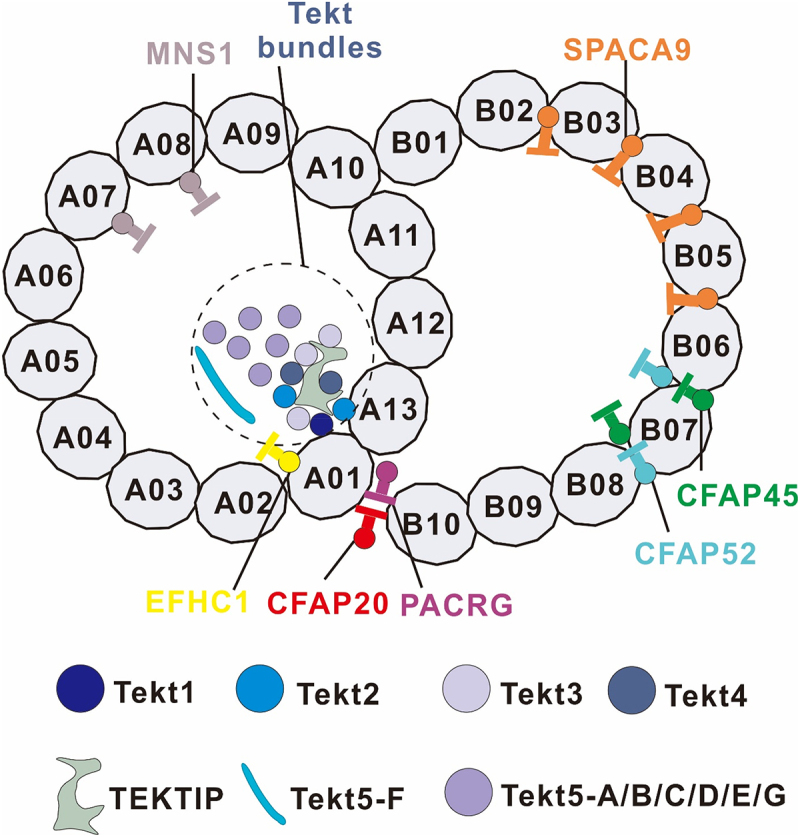
Identification of MIPs in cross-sections of the 48 nm DMT.

Beyond the prominent axonemal structures, numerous smaller proteins with yet-to-be-determined identities and roles are found associating with the outer and inner surfaces of the doublet microtubules. Cryo-ET of Chlamydomonas cilia was used to determine the structural basis of DMTs, revealing periodic high-density structures on the inner surfaces of A- & B-tubules, which were subsequently termed microtubule inner proteins (MIPs) [3]. The luminal surfaces of DMTs are highly decorated with MIPs, which are important for DMT stability (Table 1). MIPs are believed to stabilize the axoneme, especially to promote resistance to the mechanical stress associated with ciliary beating. Recent high-resolution structures of DMTs from motile mammalian cilia have shown that DMTs from different organisms contain species-specific MIPs [30] (Figure 1).

**Table 1.** Research findings on the effects of MIPs on the stability of DMTs.

MIPs	UniProt ID/ NCBI accession	Molecular mass in Da/(number of residues)	Ultra -structural localization	Periodi-city (nm)	Proto -filament location	Built residues	Underlying mechanisms
CFAP77 (Cilia and Flagella Associated Protein 77) [4]	A0A3Q1LJD6	32,939(284)	A-tubule and B-tubule	16	A11-A12, B01	116–1, 167–202, 216–284	Promotes the assembly of the doublet outer junction and stabilizes the doublet outer junction
CFAP106	E1B836	30,157(259)	A-tubule and B-tubule	16	A12-A13, B09	10–253	
CFAP276	E1B9I5	19,358(170)	A-tubule and B-tubule	16	A12-A01, B09-B10	66–100, 128–169	
CFAP53 [5]	F1N7G5	62,015(514)	A-tubule and B-tubule	48	A07-A10, B01	43–507	Stabilizes dynein binding to microtubules
CFAP127 [6,7](MNS1)	Q2KIQ2	60,419(495)	A-tubule and B-tubule	48	A08-A11, B01	15–484	Associates with outer dynein arms (ODAs) via its interaction with the ODA docking complex (ODA-DC) component CCDC114
CFAP141 (C1ORF189)	Q32L75	12,339(101)	A-tubule and B-tubule	48	A08-A11, B01	11–101	
CIMIP2A (FAM166A)	G3X6E2	36,204(320)	A-tubule	8/16	Connecting the PFs A01-A06 to Tekt bundle	15–57, 61–109, 176–318	
CIMIP2C (FAM166C)	Q3SZR5	23,379(201)	A-tubule	16	A13-A02	3–57, 97–193	
RIB72 [8]	A0A7M7RHU7	85,651(744)	A-tubule	16	A01-A05, A13	2–712, 730–744	Affects other A-tubule MIPs and the localization of B-tubule MIPs
RIB74	Q6TXM2	74,033(645)	A-tubule	16	A01-A05, A12-	2–368, 375–536, 557–643	Associates with the Ribbon in the lumen of the A-tubule
SPMIP10 (TEX43)	A0A7M7G0R8	14,165(119)	A-tubule	16	A11-A12	10–113	
Tekt1 [9,10]	Q26623	46,620(402)	A-tubule	16	A13-A01	2–401	Regulates ciliogenesis and ciliary motility
Tekt2 [11]	A0A7M7SXG5	49,765(430)	A-tubule	16	A12-A13	2–403	Regulates ciliogenesis and ciliary motility
Tekt3 [12]	A6H782	56,681(490)	A-tubule	16	A12-A13	67–490	Regulates ciliogenesis and ciliary motility
Tekt4 [13]	Q9U0E3	52,956(462)	A-tubule	16	A12	65–461	Regulates ciliogenesis and ciliary motility
Tekt5 [14]	F1MGH5	56,490(489)	A-tubule	16/48	Tekt bundle	91–477	Regulates ciliogenesis and ciliary motility
Tektl1 [15] (CCDC105)	A0A7M7RBY5	49,862(448)	A-tubule	16	A11	15–198, 211–446	Stabilizes the doublet outer junction
DUSP21	Q0II40	22,052(196)	A-tubule	16	Associated with Tekt bundle	19 −55, 64 −159	
EFHC1 [16] (EJM1)	E1BKH1	74,032(640)	A-tubule	16	Connecting the PFs A01 and A13 to Tekt1–4 bundles, respectively	8–54, 84–367, 414–531	Promotes ciliary axoneme formation
EFHC2	A0A3Q1N1R0	85,454(733)	A-tubule	16	A13-A05	2–188, 201–302, 418–595, 602–684	
CFAP126 (FLATTOP)	Q3SZT6	21,185(196)	A-tubule	16	A12-A13	2–118	
PIERCE1 [17]	A0A7M7GRL1	13,900(123)	A-tubule	24	A07-A08	40–117, 68–120	Regulates ciliogenesis and/or ciliary motility of the node
CFAP107 (C1ORF158)	A0A7M7RF95	272,295(235)	A-tubule	48	A09-A12	2–228	
CFAP95 (C9ORF135)	A0A7M7RBC4	25,660(220)	A-tubule	48	A09-A12	12–218	
CFAP68 (C11ORF1)	A0A7M7RBK2	17,653(153)	A-tubule	48	A09-A10	6–147	
CFAP67 [18] (NME7)	A0A7M7SX53	42,855(380)	A-tubule	48	A09	2–379	Contributes to the regulation of the microtubule-organizing center
CFAP143 [19] (SPAG8)	A0A7M7N7W6	23,135(204)	A-tubule	48	A09-A13	5–204	Interacts with microtubules and modulates microtubule assembly and activity
RIB35 (LOC578017)	A0A7M7THB5	34,915(310)	A-tubule	48	A06-A07, A13-A01	2–135, 236–310	
RIB43a [20]	A0A7M7RDQ5	45,083(379)	A-tubule	48	A01, A12-A13	1–379	The Rib43a-tubulin interaction leads to an elongated tubulin dimer distance every 2 dimers
SAXO3	A0A7M7THD0	36,975(322)	A-tubule	48	A12-A13	5–322	
SAXO4 [15]	A0A7M7P181	52,714(469)	A-tubule	48	A13-A01	3–456	Tags doublet microtubules and stabilizes the tubulin lattice
SPATA45	A0A7M7RHE9	9,853(83)	A-tubule	48	A10-A11, B01-B02	1–46, 55–83	
SPMIP2 (C4ORF45)	A0A7M7RHI6	32,424(294)	A-tubule	48	A08-A09	70–155	
SPMIP7 (SPATA48)	XP_041454576.1	22,977(200)	A-tubule	48	A07-A09	13–65, 99–191	SPMIP7 deficiency impairs spermatogenesis and leads to azoospermia
SPMIP8 (TEPP)	A0A7M7RCY5 /A0A7M7N5A5	21,385 (185) /23,592 (206	A-tubule	48	A09-A12	18–145, 98–185	
SPMIP11 (TEX49)	A0A7M7RDT3	17,456(150)	A-tubule	48	A07	1–150	
SPMIP11-like (LOC580808)	A0A7M7RFU3	20,661(179)	A-tubule	48	A07	78–178	
SPMIP12 (LOC577943)	A0A7M7RHW6	34,619(302)	A-tubule	48	A11-A12	3–301	
SAXO5 (TEX45)	A0A7M7RI36	55,023(492)	A-tubule	96	A04	6–256, 265–485	
CFAP91 [21]	A0A7M7NC68	121,045(1,081)	Inner junction (The shared region of the A- and B-tubules)	96	A01-B10	12–132	Knockdown of CFAP91 using RNAi impairs flagellar movement, leading to CPC defects in Trypanosoma
PACRG [22] (BUG21)	A0A7M7REU1	26,614(236)	Inner junction	8	A01-B10	11–235	Promotes microtubule assembly
CFAP20 [22] (BUG22)	A0A7M7SUA7	22,860(195)	Inner junction	8	A01-B10	1–187	Regulates ciliogenesis and intraflagellar transport (IFT)
SPACA9 [23] (C9orf9)	A0A3Q1MYU9	25,137(224)	B-tubule	8/48	B02-B06	1–157	Binds SAXO (stabilizer of axonemal microtubules) proteins to maintain the stability of B-tubules
EFCAB6 [24]	XP_003586209.2	172,744(1,497)	B-tubule	16	B10	1319–1496	Highly associated with the presence of motile ciliated cells within human tissue
CIMIP3 (MGC137036)	A0A3Q1LFG5/ Q2NKT6	21,204(192)	B-tubule	16	B10	139–192	
CFAP52 [25,26] (HTX10)	A0A7M7NV05	70,362(635)	B-tubule	16	B06-B08	19–633	Changes dynein-driven microtubule sliding via an adenine nucleotide homeostasis module
CFAP21 [27] (EFHB)	A0A7M7RFU1	66,077(592)	B-tubule	48	A08-A09	25–416, 447–589	Engages in flagellar assembly and sperm motility, fertilization, and male gametogenesis
CIMIP1 (C20ORF85)	A0A7M7HL88	23,698(206)	B-tubule	48	B07-B08	11–68	
CIMIP4 [28] (TEX33)	A0A7M7NRA7	40,476(359)	B-tubule	48	B07-B09	252–352	Regulates manchette (a specialized type of non-centrosomal microtubule that encircles the head of elongated spermatid) morphology and spermiogenesis in association with cooperating factors
CFAP90	A0A7M7SYJ8	19,673(171)	B-tubule	48	B09-B10	70–164	
CFAP144 [24] (FAM183A)	A0A7M7GGZ7	16,406(139)	B-tubule	48	B07-B08	13–60, 66–136	Highly associated with the presence of motile ciliated cells within human tissue
CFAP210 (CCDC173)	A0A7M7REW2	65,653(560)	B-tubule	48	B06-B10	44–560	
SPMIP1 (ATP6V1FNB)	A0A7M7GIN5	21,930(188)	B-tubule	48	B07-B10	2–35, 104–187, 147–187	
SPMIP13 (LOC105439830)	A0A7M7NFX5	17,287(142)	A-tubule	48	B02-B06	2–31, 96–141	
CFAP45 [29] (HTX11)	A0A7M7NFL8	63,990(541)	B-tubule	48	B06-B08	68–541	Promotes structural stability between the B-tubule protofilaments

Table 1 Comprehensive Research Findings on the Effects of MIPs on the Stability of DMTs. This table delineates the specific characteristics and functions of various MIPs within the cellular framework. Each entry spans across multiple columns, detailing the MIPs’ names, their unique UniProt ID or NCBI accession numbers, molecular mass in Daltons along with the number of residues, ultra-structural localization, periodicity in nanometers, proto-filament location, built residues, and the underlying mechanisms of their action. The table is organized to present a clear view of how each MIP contributes to the stability and functionality of DMTs, with specific mention of their roles in assembly, stabilization, and regulation of ciliary motility and axoneme formation. This compilation of data serves as a valuable resource for understanding the intricate relationships between MIPs and DMTs in cellular biology.

The term “Built residues” refers to the amino acid units that constitute the protein structure; CIMIP1, Ciliary microtubule inner protein 1; CIMIP2A, Ciliary microtubule inner protein 2A; CIMIP2C, Ciliary microtubule inner protein 2C; CIMIP3, Ciliary microtubule inner protein 3; CIMIP4, Ciliary microtubule inner protein 4; DUSP21, Dual specificity phosphatase 21; EFHC1, EF-hand domain containing 1; EFHC2, EF-hand domain containing 2; EFCAB6, EF-hand calcium binding domain 6; PACRG, Parkin coregulated; RIB35, Flagellar protofilament ribbon protein 35; RIB43a, Flagellar protofilament ribbon protein 43a; RIB72, Flagellar protofilament ribbon protein 72; RIB74, Flagellar protofilament ribbon protein 74; SAXO, stabilizer of axonemal microtubules; SAXO3, Stabilizer of axonemal microtubules 3; SAXO4, Stabilizer of axonemal microtubules 4; SAXO5, Stabilizer of axonemal microtubules 5; SPATA45, SPACA9, Sperm acrosome associated 9; SPATA45, Spermatogenesis associated 45; SPMIP2, Sperm microtubule inner protein 2; SPMIP7, Sperm microtubule inner protein 7; SPMIP8, Sperm microtubule inner protein 8; SPMIP10, Sperm microtubule inner protein 10; SPMIP11, Sperm microtubule inner protein 11; Tekt1, Tektin1; Tekt2, Tektin2; Tekt3, Tektin3; Tekt4, Tektin4; Tekt5, Tektin5; Tektl1, Tektin like 1;

Tektin bundles are an important class of MIPs in the filamentous protein family. Tektin proteins are insoluble in water [Reference: Linck, R.W. and Langevin, G.L. (1982) Structure and chemical composition of insoluble filamentous components of sperm flagellar microtubules. J. Cell Sci., 58(1), 1–22]. Their protein sequences contain repeats of a conserved peptide of nine amino acids that may bind tubulin [31]. Tektin proteins (Tekts) interact with other MIPs and microtubule proteins in the A-tubule of the DMT to form ciliated axonemes. Tekts, ubiquitous among eukaryotes, are essential for the integrity and function of cilia and flagella. They form α-helical structures that assemble into continuous rods approximately 2 nm in diameter, providing a key stabilizing framework for microtubules [32]. The protein bundle, which is composed of assembled Tekt1–5 protein filaments, strengthens the DMTs by increasing their rigidity and stability. This reinforcement optimizes the substrate structure, enabling more efficient force generation during ciliary motion. In this review, we focus on the biological function of Tekt bundles, their roles in diseases, and the possible underlying mechanisms.

## Biological functions of Tekt bundles

Tekts are filament-forming proteins that coassemble with tubulins to form axonemal microtubules, basal bodies, and centrioles in cilia and flagella. The Tekt family of proteins is evolutionarily conserved across eukaryotes and forms hetero- and homo-dimers via coiled-coil domains with the ribbon filaments of DMTs. Tekt expression has been reported in the testis, brain, retina, and other tissues containing ciliated cells [9].

Tekts constitute a family of microtubule-stabilizing proteins, with five identified members in mammals that are essential for the assembly and motility of cilia and flagella. First, Tomomi Nishie and colleagues reported that during the 5–8 week differentiation period of embryoid bodies (EBs) from cynomolgus monkeys, Tekt1 expression gradually increases in several tissues containing ciliated epithelium (lungs, trachea, fallopian tubes, and testes). After eight weeks of EB differentiation, 9+2 microtubule structures can be observed in ciliated epithelial tissues [33]. Rebecca Ryan et al. confirmed that Tekt1 is present at the basal body of primary cilia (as previously indicated). Variations in Tekt1 result in decreased centrosomal localization in affected individuals. Recent studies have highlighted the importance of these genes in Drosophila development and function [9]. Specifically, Drosophila Tekt genes have been implicated in essential functions, with null mutants being lethal at the late pupal stage, indicating a critical role in development. Additionally, tissue-specific knock-down studies have revealed that Tekt function is required for larval locomotion, ovary development, and control of adult circadian rhythm in Pigment Dispersing Factor neurons. Tet protein function during Drosophila development

Similarly, Tekt1 is present at the axoneme of motile cilia. When Tekt1 expression is knocked down, zebrafish presented very mild body curvature, a classical ciliopathy-associated phenotype thought to be linked to convergence–extension defects. Furthermore, the reduced motility of cilia in Tekt1 morphants was further confirmed by measuring the angle of motility formed by beating cilia in Kupffer’s vesicle (KV) [9]. The study demonstrated that Tekt1 expression in pronephros depends on Foxj1a and Foxj1b. Moreover, Foxj1a/b double knockdown consistently abolished the expression of Tekt1. It impaired the ciliary motility of zebrafish embryos, resulting in immobile cilia in KV [34]. Taken together, these findings indicate that Tekt1 is not only required for motile cilia in various organs but also may act as a positive regulator of primary cilia length. The aforementioned findings indicate that Tekt1 not only is crucial for the effective motility of motile cilia but also likely plays a role in regulating the length of primary cilia.

Several studies have demonstrated that Tekt2 maintains the integrity of the ciliary structure, normal spermatogenesis, and sperm motility. Using Gene Expression Omnibus datasets, Haiming Cao and colleagues reported that Tekt2 was downregulated in the context of abnormal spermatogenesis (based on analysis of GSE45885, GSE45887 and GSE9210), and related to azoospermia in male infertility (based on analysis of GSE4797, GSE145467, GSE108886, and GSE6872) [35]. Eiman Alshawa et al. reported a significant reduction in the expression level of Tekt2 in cryopreserved samples compared with fresh samples, which could indicate its potential use as a marker of sperm motility loss [36]. In a study of an appropriate zebrafish model for early-onset retinal degeneration, Danna Jia et al. reported on the effects of Tulp1 mutation in zebrafish, observing changes in Tekt2 expression levels. While these data suggest a potential link between Tekt2 and the observed phenotypes, it is important to note that the study does not establish a direct causal relationship. The findings from Jia et al. indicate that Tekt2 may be associated with the processes impacting photoreceptor survival, but further research is needed to determine the precise role of Tekt2 in these biological outcomes [11]. These findings suggest that Tekt2 plays a significant role in spermatogenesis and may serve as a potential biomarker for the loss of sperm motility, as well as being crucial for the survival of photoreceptors.

Tekt3 promote sperm progressive motility by interacting with Tekt1, 2, 4, and 5 to form a multimeric protein complex. Hiroe Takiguchi and colleagues reported that Tekt3 is not only required for flagellar stability and sperm motility, but it may also be involved in acrosome-related events, such as sperm‒egg fusion [37]. Yiyuan Liu et al. identified three mutations in the TEKT3 gene in 100 males with asthenozoospermia. The mutations include a homozygous deletion-insertion mutation (c.543_547delinsTTGAT: p.Glu182*) and a compound heterozygous mutation (c.[548G > A]; [752A > C], p.[Arg183Gln]; [Gln251Pro]). suggesting that loss of Tekt3 function may lead to oligoasthenoteratozoospermia in humans. Moreover, Mice lacking Tekt3 exhibit reduced sperm motility and structural defects in sperm flagella, which aligns with the observed phenotype in our human patients harboring TEKT3 mutations [12]. A study has revealed that that male mice lacking Tekt3 produce sperm with reduced motility and forward progression as well as increased flagellar structural bending defects [38]. Xin-Yan Geng et al. suggested that the loss of the Tekt3-interacting protein TEKTIP affects the native conformation of Tekt3, which reduces the abundance of Tekt3-high polymers and increases the abundance of its oligomeric form and interactions with other Tekts. More Tekt3 heterodimers and oligomers further influence the integrity and stability of Tekt bundles, leading to partial disorganization of the sperm flagella axoneme, altering sperm motility, and promoting male subfertility [39]. These findings underscore the pivotal role of TEKT3 in sperm motility and fertility.

Tekt4 is required for progressive sperm motility. Angshumoy Roy et al. reported that the fertility of Tekt3^−/−^; Tekt4^−/−^ double-knockout males was significantly lower than that of Tekt3^−/−^ single-knockout males or WT males [38]. The authors also demonstrated that Tekt4-null sperm exhibit drastically reduced forward velocity and uncoordinated waveform propagation, leading to excessive consumption of ATP along the flagellum. The absence of Tekt4 causes asthenozoospermia and subfertility in male mice [40]. A recent investigation has shown that in MDA-MB-468 cells, expression of a variant Tekt4 reduces the level of acetylated tubulin (which promotes microtubule stability) and that polymerized tubulin deregulates microtubule stability and antagonizes the paclitaxel-induced stabilizing effect on microtubules and increases paclitaxel resistance. The stabilizing effect of Mut Tekt4 on microtubules is weaker than that of WT Tekt4 [41]. In summary, Tekt4 is pivotal in regulating sperm motility and fertility. Its loss of function leads to diminished sperm viability and reduced male fertility. Moreover, Tekt4 plays a crucial role in microtubule stability and breast cancer sensitivity to drugs.

Recent studies have demonstrated that Tekt5 contributes to spermiogenesis by maintaining the level of acetylated α-tubulin, which is critical for microtubule stability. Researchers have discovered heterozygous deletions in the Tekt5 gene in an individual with azoospermia using array-based comparative genomic hybridization (aCGH) [42]. Nana Aoki et al. reported that the expression of the Tekt5 protein is gradually upregulated in the testis from the late pachytene stage during spermatogenesis. Knockdown of Tekt5 in the testis resulted in a significant decrease in the number of spermatids and a reduction in the level of acetylated α-tubulin in vivo [43]. These studies suggest that Tekt5 is essential for sperm development and maturation, and also promotes spermatogenesis by maintaining appropriate levels of acetylated α-tubulin, a critical determinant of microtubule stability as well as sperm structure and function. Consequently, Tekt5 expression and function significantly impact sperm production and motility. These insights into the molecular mechanisms of spermatogenesis may offer novel targets for the treatment of male infertility.

In essence, the Tekt1, Tekt2, Tekt3, Tekt4, and Tekt5 proteins are integral components of internal microtubule bundles in the sperm flagellum axoneme and are indispensable for sperm motility. They stabilize cilia and flagella by assembling into resilient polymeric complexes. Their fundamental role is to support the structural integrity and motility of cilia and flagella. Despite their varied functions in different tissues and cells, these proteins uniformly underpin the structural integrity and motility of cilia and flagella. Their conserved function involves facilitating ciliary movement through the formation of internal microtubule structures. Individual Tekt proteins may also play distinct roles in specific biological processes, such as Tekt2 in photoreceptor survival, Tekt4 in breast cancer, and Tekt5 in sperm formation and cancer cell survival.

## Tekt bundles and diseases

The luminal surfaces of ribbon filaments are highly decorated with Tekt bundles, which are important for DMT stability. Abnormalities in Tekt protein structure or expression cause infertility, tumours, and ciliopathies, including retinal degeneration, MZSDS, and diabetic nephropathy (DN).

### Infertility

Zhaocheng Xiong et al. reported that the expression level of Tekt2 in Murrah buffalo sperm is highly positively correlated with sperm motility [44]. A study reported a heterozygous mutation in Tekt2 in a nonsyndromic asthenozoospermia patient. Ultrastructural analysis revealed anomalies involving axoneme microtubules and mitochondria in ≥80% of the examined spermatozoa [45]. A study has indicated that inhibition of tyrosine phosphorylation of Tekt is associated with impaired flagellar bending and motility in hamster spermatozoa [46]. Zhao et al. reported that Tekt2 was associated with distal droplets (a phenotype of abnormal sperm morphology) in a Duroc boar population via a weighted single-step GWAS (wssGWAS) [47]. Angshumoy Roy and colleagues suggested that the time-dependent decrease in progressive motility in Tekt4-null sperm and the structural defects in Tekt3-null sperm might additively reduce the fertility potential of these sperm [38]. Research has shown that Ccdc38 knockout causes acrosomal hypoplasia and impairs sperm motility by downregulating Tekt3 [48]. Ashok Agarwal et al. reported significantly impaired sperm morphology, elevated levels of ROS, and overexpression of Tekt3 in a bilateral varicocele group [49]. Zahid Parvez Sukhan et al. demonstrated that Tekt4 mRNA expression was upregulated during the fully mature testicular developmental stage of seasonal development. Furthermore, the mRNA expression of Tekt4 was significantly greater in sperm with greater motility than in those with lower motility during the peak breeding season, during the induced spawning activity stages, and after cryopreservation in different cryoprotectants [13]. New findings reveal that sperm extracted from Tekt5 knockout mice had a lower percentage of motile cells than those extracted from WT controls and a greater percentage of cells with defective flagella with 180° bends [14].

Summar Sohail and colleagues reported that Tekt1 knockdown increased male sterility in fruit flies by affecting the production of mature spermatozoa and their viability [50]. A series of studies have implicated that abnormally elevated miR-199-5p expression inhibited sperm flagella formation during spermiogenesis by negatively regulating the expression of Tekt1, causing sperm abnormalities in male allotriploid crucian carp [51]. Another study reported that Tekt2 was significantly downregulated in nonobstructive azoospermia. Through bioinformatic analysis and experimental studies, the TEKT2 gene was identified as a central hub for impaired spermatogenesis resulting from NOA, testicular germ cell tumours, and orchitis [52]. A separate study reported that the number of spermatids was significantly decreased and that the level of acetylated α-tubulin was decreased in vivo by knockdown of Tekt5 in the testis [43]. The data presented here reveal a correlation between infertility and alterations in Tekt proteins. These findings suggest a significant link between Tekt2 expression levels and sperm motility. Tekt2 mutations are associated with axoneme microtubule abnormalities, which can lead to reduced sperm motility. Additionally, this study highlights the important roles of Tekt3 and Tekt4 in influencing both sperm motility and overall fertility. These proteins appear to play crucial roles in maintaining the structural integrity and function of the sperm, which are essential for successful fertilization.

### Tumours

Research has shown that breast cancer cells harboring Tekt4 germline mutations were associated with resistance to paclitaxel-based chemotherapy by stabilizing the tubulin structure in doublet microtubules [53]. Breast cancer cells with these Tekt4 variations are relatively sensitive to the antitumour drug vinorelbine (a microtubule-depolymerizing agent) [53]. Furthermore, they reported that Tekt4 germline variations were enriched in posttreatment breast cancer tissues. Compared with wild-type Tekt4, Tekt4 germline mutations are associated with reduced disease-free survival and overall survival in patients receiving paclitaxel-based chemotherapy [41]. Further studies have indicated that TEKT4 knockdown inhibits thyroid tumourigenesis by impairing cell proliferation, colony formation, migration, and invasion [54]. Loss of Tekt4 has also been linked to increased migration, invasion, and metastasis in triple-negative breast cancer cells, as well as decreased microtubule stability due to enhanced interaction between HDAC6 and α-tubulin [53]. In ovarian cancer cells, Tekt5 knockdown has been shown to induce G1 arrest and apoptosis by upregulating p27kip1 (a cyclin-dependent kinase inhibitor) [43]. These findings indicate that whether Tekt4 inhibits or promotes tumour invasion depends on the tumour type and subtype, and that the presence of Tekt4 germline mutations can lead to resistance to chemotherapy in breast cancer and other tumours. These mutations are linked to reduced survival rates among patients receiving paclitaxel-based chemotherapy. Furthermore, the role of Tekt4 in thyroid tumourigenesis and the influence of Tekt5 on cell cycle regulation in ovarian cancer cells highlight a significant connection between Tekt proteins and the development of cancer. This association underscores the potential impact of Tekt proteins on tumour biology and the response to cancer treatments.

### Ciliopathies

Studies have revealed that a child who presented with MZSDS, characterized by renal, retinal, and skeletal involvement, was also diagnosed with lung infections and airway ciliary dyskinesia. A translation-blocking morpholino for Tekt1 resulted in the loss of Tekt1 and a ciliopathy phenotype with situs inversus and cysts in the pronephros [9]. Research has also shown that the mRNA level of Tekt2 was negatively correlated with the mRNA levels of NPHS1 (a member of the immunoglobulin family of cell adhesion molecules that functions in the glomerular filtration barrier in the kidney) and NPHS2 (similar to NPHS1) in human diabetic glomeruli. Immunostaining confirmed that the expression of Tekt2 was increased in the podocytes of DN patients and diabetic mice. Knocking down Tekt2 resulted in resistance to high-glucose-induced cytoskeletal remodeling and downregulation of the NPHS1 protein in cultured podocytes [55]. Overall, these observations indicate that the cytoskeletal regulatory gene TEKT2 is involved in the cytoskeletal remodeling of podocytes, contributing to the pathogenesis of DN. Danna Jia et al. reported that the loss of TULP1 causes defects in ciliary structure and opsin trafficking by downregulating Tekt2, further increasing the death of photoreceptors via ferroptosis [11]. This research reveals a link between ciliopathies, such as Mainzer-Saldino syndrome and diabetic nephropathy, and the dysfunction of Tekt proteins. Specifically, Tekt1 and Tekt2 have been implicated in the pathogenesis of these diseases. Tekt1 is associated with ciliary dyskinesia, whereas Tekt2 plays a role in cytoskeletal remodeling within podocytes. These functions are crucial for maintaining cellular integrity and, when disrupted, can lead to the development of the aforementioned ciliopathies. These findings underscore the importance of Tekt proteins in cellular health and disease progression.

In conclusion, the role and significance of Tekt protein bundles in diseases are multifaceted, encompassing conditions such as infertility, cancer, and ciliopathies. Delving deeper into the study of these proteins not only aids in our comprehension of the pathogenesis of these diseases but also holds the potential to uncover vital clues for the development of novel therapeutic strategies.

## Mechanisms of Tekt protein regulation

Pathophysiological stimuli can regulate Tekt bundles at the transcriptional, posttranscriptional (splicing variants), translational, and posttranslational (phosphorylation and deacetylations) levels. Nathan E Hellman et al. reported that increased Foxj1a expression in obstructed tubules induced the ciliary motility target gene TEKT1 expression. In Foxj1a -deficient embryos, Tekt1 cannot be upregulated, and increased ciliary beat rates cannot be maintained after obstruction [34]. Rebecca Ryan et al. reported the identification of compound heterozygous variations in Tekt1. One Tekt1 variation corresponded to a rare nonsense variant predicted to lead to the synthesis of a truncated protein with loss of the last two C-terminal coiled coils [9]. Through bioinformatics analysis, a putative miR-199-5p binding site in Tekt1 mRNA was identified. Further, via a luciferase reporter assay, Tekt1 was confirmed as a target of miR-199-5p. Tulp1a and Tulp1b were reported to act as transcription factors to promote the expression of Tekt2 to induce ciliogenesis [51]. A functional association was reported that Tulp1a and Tulp1b act as transcription factors to promote the expression of Tekt2 to induce ciliogenesis [11]. Daniel Mariappa and colleagues reported the functional association of the hypo tyrosine phosphorylation status of Tekt2 with impaired flagellar bending of spermatozoa following the inhibition of EGFR tyrosine kinase [46]. A study demonstrated that the knockout of CCDC38 (a coiled-coil domain-containing protein that is required for acrosome biogenesis and fibrous sheath assembly) resulted in a decreased level of Tekt3 in the testis and an aberrant distribution of Tekt3 in sperm [56]. Hyperactivation of bull spermatozoa by cell-permeable cAMP and calyculin A, a protein phosphatase inhibitor, promoted the translocation of Tekt3 from the post-acrosomal region to the middle segment in sperm heads and that Tekt3 accumulation at the middle segment was lost upon the acrosome reaction [57]. An examination of the frequency of enriched Tekt variations in an independent extension cohort of 84 paired samples of different molecular subtypes revealed that most of the enriched variations in the Tekt family members were in Tekt4. By Sanger sequencing, 28 variations, including four in Tekt1 (14.3%, two cases), two in Tekt2 (7.1%, one case), two in Tekt3 (7.1%, one case), 16 in Tekt4 (57.2%, eight cases) and four in Tekt5 (14.3%, two cases), were identified in 60 breast cancer samples. Most of the enriched variations in the Tekt family members were in Tekt4. Further sequencing confirmed the Tekt4 status in an additional 24 breast cancer samples and revealed that after NCT, the original genotype was maintained in 74 cases (88.1%), whereas the Tekt4 variations c. A541G and c. A547G was enriched in 10 patients (11.9%). Among the ten patients, two had the luminal A subtype, one had the luminal B subtype, one was HER2+, and six had BLBC. Taken together, these observations indicate that these two variations, which were initially identified in tumour tissues, are common (each with a minor allele frequency >10%) germline polymorphisms rather than low-frequency somatic changes [41]. Researchers have identified a novel microtubule-associated complex containing Tektin4 and histone deacetylase 6 (Hdac6). Tekt4 bound to the DAC1 domain of HDAC6 via residues 218–326. Tektin4 loss increased the interaction between HDAC6 and α-tubulin, thus decreasing microtubule stability through HDAC6-mediated tubulin deacetylation [58]. A study reported that the top GWAS hits associated with all-cause mortality among people with coronary artery disease included 1 SNP (rs9932462, Tekt5) among 510 males [59]. Another study revealed that whole exome sequencing (WES) data from the MERGE study, which included 2,030 men, revealed variants that might affect allele assignment in the TEKT5 gene in four men [42]. In summary, FOXJ1A can promote transcription of the TEKT1 gene, whereas Tulp1a/b can promote transcription of the TEKT2 gene. Pre-mRNA mutations in Tekt1, Tekt4, and Tekt5 are strongly correlated with the occurrence of civil dyskinesia, breast cancer, and coronary artery disease, respectively. MiR-199-5p can inhibit the translation of TEKT1 mRNA and posttranslational modifications of Tekt2/3, such as the modification of Tekt2/3 with low phosphorylation, which can cause damage to sperm flagellar curvature and the translocation of Tekt3 from the acrosome to the middle segment of sperm. Hdac6 can also compete with Tekt4 for α-tubulin binding, mediating microtubule deacetylation, and reducing microtubule stability.

In summary, this review highlights the complex regulation of Tekt proteins in response to various pathophysiological signals. Regulation of Tekt1, Tekt2, Tekt3, Tekt4, and Tekt5 proteins spans several layers, including transcriptional activation by FOXJ1A and Tulp1a/b, posttranscriptional modifications mediated by miR-199-5p, and posttranslational modifications such as phosphorylation and deacetylation. The intricate interplay between Tekt4 and HDAC6 in modulating microtubule stability, along with genetic variations in TEKT1, TEKT4, and TEKT5, which are associated with diseases, further underscores the sophisticated regulatory mechanisms that govern the function and activity of Tekt proteins. These insights into the multilevel control of Tekt proteins provide a deeper understanding of their role in health and disease.

## Conclusion and perspectives

The primary function of Tekt bundles may be to maintain the stability of DMTs and the motility of cilia and flagella. Abnormalities in Tekt are among the most common pathophysiological features of many diseases associated with microtubule instability. As indicated by the results of the studies discussed in this review of the recent literature, pathophysiological stimuli can regulate the expression of Tekt bundles through transcriptional, posttranscriptional (e.g., splice variants), translational (e.g., miRNA), and posttranslational (e.g., phosphorylation) mechanisms.

Recent advancements in Tekt protein research have opened new directions for future studies. For example, therapeutic strategies targeting Tekt proteins could increase the fertility of patients with infertility or improve the response of cancer patients to chemotherapy. Further research into the role of Tekt proteins in ciliopathies may reveal new therapeutic targets. The implications of these findings for past work mean that our understanding of the role of Tekt proteins in fertility, cancer, and ciliopathies has significantly expanded. This new knowledge can lead to a reevaluation of existing treatments and the development of more targeted interventions.

For future work, it would be beneficial to focus on the (i) detailed mechanisms of Tekt protein action, (ii) their specific roles in various diseases, and how their functions can be modulated through (iii) pharmaceutical interventions. (iv) Mechanistic Studies delve deeper into the biochemical and biophysical properties of Tekt proteins, including their interactions with other cellular components and their impact on cellular processes such as cell division, motility, and signaling. (v) Disease-Specific Roles: examine the specific contributions of different Tekt proteins to disease pathology. For example, how do mutations in Tekt4 lead to resistance to chemotherapy in breast cancer, and what are the mechanisms by which Tekt proteins influence thyroid tumourigenesis and ovarian cancer cell cycle regulation? (vi) Therapeutic Interventions: develop and test potential drugs that modulate Tekt protein function. This could include small molecules that bind to Tekt proteins and alter their activity or stability or biologics that target Tekt protein expression or localization. (vii) Personalized Medicine: investigate the potential for personalized medicine approaches using Tekt protein profiles. For example, could genetic variations in Tekt proteins be used to predict an individual’s response to certain treatments, such as chemotherapy? (viii) Ciliopathies Research: expand research on the role of Tekt proteins in ciliopathies, exploring how dysfunction of these proteins contributes to the development of diseases such as Mainzer-Saldino syndrome and diabetic nephropathy. Understanding these mechanisms could lead to new treatments for these conditions.

In conclusion, the future of Tekt protein research looks promising, with the potential to significantly impact our understanding and treatment of a range of diseases. By focusing on the areas outlined above, researchers can build on recent advancements to uncover new insights and develop more effective therapies.

## Data Availability

Data sharing is not applicable to this article as no new data were created or analyzed in this study.

## Abbreviations

aCGH, array-based comparative genomic hybridization; CFAP, Cilia and Flagella Associated Protein; DMT, Doublet microtubule; DMTs, Doublet microtubules; DN, Diabetic nephropathy; EBs, Embryoid bodies; IFT, Intraflagellar transport; KV, Kupffer’s vesicle; MIPs, Microtubule inner proteins; MZSDS, Mainzer–Saldino syndrome; N-DRC, Nexin–dynein regulatory complex; ODAs, Outer dynein arms; PCD, Primary ciliary dyskinesia; PFs, Protofilaments; Tekt, Tektin; Tekts, Tektin proteins; wssGWAS, weighted single-step GWAS; WES, Whole exome sequencing

## References

[1] Reiter JF, Leroux MR. Genes and molecular pathways underpinning ciliopathies. Nat Rev Mol Cell Biol. 2017;18(9):533–12. doi: 10.1038/nrm.2017.60 28698599 PMC5851292

[2] Klena N, Pigino G. Structural Biology of Cilia and Intraflagellar Transport. Annu Rev Cell Dev Biol. 2022;38(1):103–123. doi: 10.1146/annurev-cellbio-120219-034238 35767872

[3] Nicastro D, Fu X, Heuser T, et al. Cryo-electron tomography reveals conserved features of doublet microtubules in flagella. Proc Natl Acad Sci USA. 2011;108(42):E845–53. doi: 10.1073/pnas.1106178108 21930914 PMC3198354

[4] Kubo S, Black CS, Joachimiak E, et al. Native doublet microtubules from Tetrahymena thermophila reveal the importance of outer junction proteins. Nat Commun. 2023;14(1):2168. doi: 10.1038/s41467-023-37868-0 37061538 PMC10105768

[5] Willekers S, Tessadori F, van der Vaart B, et al. The centriolar satellite protein Cfap53 facilitates formation of the zygotic microtubule organizing center in the zebrafish embryo. Development. 2022;149(16). doi: 10.1242/dev.198762 PMC9481976 35980365

[6] Zhou J, Yang F, Leu NA, et al. MNS1 is essential for spermiogenesis and motile ciliary functions in mice. PLOS Genet. PLOS Genet. 2012;8(3):e1002516. doi: 10.1371/journal.pgen.1002516 22396656 PMC3291534

[7] Ta-Shma A, Hjeij R, Perles Z, et al. Homozygous loss-of-function mutations in MNS1 cause laterality defects and likely male infertility. PLOS Genet. 2018;14(8):e1007602. doi: 10.1371/journal.pgen.1007602 30148830 PMC6128653

[8] Fabritius AS, Bayless BA, Li S, et al. Proteomic analysis of microtubule inner proteins (MIPs) in Rib72 null Tetrahymena cells reveals functional MIPs. Mol Biol Cell. 2021;32(21):br8. doi: 10.1091/mbc.E20-12-0786 34406789 PMC8693976

[9] Ryan R, Failler M, Reilly ML, et al. Functional characterization of tektin-1 in motile cilia and evidence for TEKT1 as a new candidate gene for motile ciliopathies. Hum Mol Genet. 2018;27(2):266–282. doi: 10.1093/hmg/ddx396 29121203

[10] Leung MR, Zeng J, Wang X, et al. Structural specializations of the sperm tail. Cell. 2023;186(13):2880–2896 e17. doi: 10.1016/j.cell.2023.05.026 37327785 PMC10948200

[11] Jia D, Gao P, Lv Y, et al. Tulp1 deficiency causes early-onset retinal degeneration through affecting ciliogenesis and activating ferroptosis in zebrafish. Cell Death Dis. 2022;13(11):962. doi: 10.1038/s41419-022-05372-w 36396940 PMC9672332

[12] Liu Y, Li Y, Meng L, et al. Bi-allelic human TEKT3 mutations cause male infertility with oligoasthenoteratozoospermia owing to acrosomal hypoplasia and reduced progressive motility. Hum Mol Genet. 2023;32(10):1730–1740. doi: 10.1093/hmg/ddad013 36708031

[13] Sukhan ZP, Hossen S, Cho Y, et al. Hdh-Tektin-4 Regulates Motility of Fresh and Cryopreserved Sperm in Pacific Abalone, Haliotis discus hannai. Front Cell Dev Biol. 2022;10:870743. doi: 10.3389/fcell.2022.870743 35547812 PMC9081794

[14] Chen Z, Shiozaki M, Haas KM, et al. De novo protein identification in mammalian sperm using in situ cryoelectron tomography and AlphaFold2 docking. Cell. 2023;186(23):5041–5053 e19. doi: 10.1016/j.cell.2023.09.017 37865089 PMC10842264

[15] Nguyen TTT, Tokuhiro K, Shimada K, et al. Gene-deficient mouse model established by CRISPR/Cas9 system reveals 15 reproductive organ-enriched genes dispensable for male fertility. Front Cell Dev Biol. 2024;12:1411162. doi: 10.3389/fcell.2024.1411162 38835510 PMC11148293

[16] Zhao Y, Shi J, Winey M, et al. Identifying domains of EFHC1 involved in ciliary localization, ciliogenesis, and the regulation of Wnt signaling. Dev Biol. 2016;411(2):257–265. doi: 10.1016/j.ydbio.2016.01.004 26783883 PMC4892117

[17] Sung YH, Baek I-J, Kim YH, et al. PIERCE1 is critical for specification of left-right asymmetry in mice. Sci Rep. 2016;6(1):27932. doi: 10.1038/srep27932 27305836 PMC4917697

[18] Sedova L, Buková I, Bažantová P, et al. Semi-lethal primary ciliary dyskinesia in rats lacking the Nme7 gene. Int J Mol Sci. 2021;22(8):3810. doi: 10.3390/ijms22083810 33916973 PMC8067621

[19] Li R, Tang X-L, Miao S-Y, et al. Regulation of the G2/M phase of the cell cycle by sperm associated antigen 8 (SPAG8) protein. Cell Biochem Funct. 2009;27(5):264–268. doi: 10.1002/cbf.1574 19548270

[20] Ichikawa M, Khalifa AAZ, Kubo S, et al. Tubulin lattice in cilia is in a stressed form regulated by microtubule inner proteins. Proc Natl Acad Sci USA. 2019;116(40):19930–19938. doi: 10.1073/pnas.1911119116 31527277 PMC6778249

[21] Martinez G, Beurois J, Dacheux D, et al. Biallelic variants in MAATS1 encoding CFAP91, a calmodulin-associated and spoke-associated complex protein, cause severe astheno-teratozoospermia and male infertility. J Med Genet. 2020;57(10):708–716. doi: 10.1136/jmedgenet-2019-106775 32161152

[22] Chen Z, Li M, Zhu H, et al. Modulation of inner junction proteins contributes to axoneme differentiation. Proc Natl Acad Sci USA. 2023;120(30):e2303955120. doi: 10.1073/pnas.2303955120 37463209 PMC10372625

[23] Gui M, Croft JT, Zabeo D, et al. SPACA9 is a lumenal protein of human ciliary singlet and doublet microtubules. Proc Natl Acad Sci USA. 2022;119(41):e2207605119. doi: 10.1073/pnas.2207605119 36191189 PMC9564825

[24] Patir A, Fraser AM, Barnett MW, et al. The transcriptional signature associated with human motile cilia. Sci Rep. 2020;10(1):10814. doi: 10.1038/s41598-020-66453-4 32616903 PMC7331728

[25] Wu B, Li R, Ma S, et al. The cilia and flagella associated protein CFAP52 orchestrated with CFAP45 is required for sperm motility in mice. J Biol Chem. 2023;299(7):104858. doi: 10.1016/j.jbc.2023.104858 37236356 PMC10319328

[26] Dougherty GW, Mizuno K, Nöthe-Menchen T, et al. CFAP45 deficiency causes situs abnormalities and asthenospermia by disrupting an axonemal adenine nucleotide homeostasis module. Nat Commun. 2020;11(1):5520. doi: 10.1038/s41467-020-19113-0 33139725 PMC7606486

[27] Greither T, Dejung M, Behre HM, et al. The human sperm proteome—Toward a panel for male fertility testing. Andrology. 2023;11(7):1418–1436. doi: 10.1111/andr.13431 36896575

[28] Xia M, Xia J, Niu C, et al. Testis-expressed protein 33 is not essential for spermiogenesis and fertility in mice. Mol Med Rep. 2021;23(5). doi: 10.3892/mmr.2021.11956 PMC7974414 33760102

[29] Owa M, Uchihashi T, Yanagisawa H-A, et al. Inner lumen proteins stabilize doublet microtubules in cilia and flagella. Nat Commun. 2019;10(1):1143. doi: 10.1038/s41467-019-09051-x 30850601 PMC6408466

[30] Ma M, Stoyanova M, Rademacher G, et al. Structure of the decorated ciliary doublet microtubule. Cell. 2019;179(4):909–922 e12. doi: 10.1016/j.cell.2019.09.030 31668805 PMC6936269

[31] Amos LA. The tektin family of microtubule-stabilizing proteins. Genome Biol. 2008;9(7):229. doi: 10.1186/gb-2008-9-7-229 PMC2530864 18671835

[32] Budamagunta MS, Guo F, Sun N, et al. Production of recombinant human tektin 1, 2, and 4 and in vitro assembly of human tektin 1. Cytoskeleton (Hoboken). 2018;75(1):3–11. doi: 10.1002/cm.21418 29108134 PMC5771813

[33] Nishie T, Ohta Y, Shirai E, et al. Identification of TEKTIN1-expressing multiciliated cells during spontaneous differentiation of non-human primate embryonic stem cells. Genes to Cells. 2023;28(7):516–525. doi: 10.1111/gtc.13031 37186436

[34] Hellman NE, Liu Y, Merkel E, et al. The zebrafish foxj1a transcription factor regulates cilia function in response to injury and epithelial stretch. Proc Natl Acad Sci USA. 2010;107(43):18499–18504. doi: 10.1073/pnas.1005998107 20937855 PMC2972951

[35] Cao H, Wan Z, Wang F, et al. Downregulation of KIF2C and TEKT2 is associated with male infertility and testicular carcinoma. Aging (Albany NY). 2021;13(19):22898–22911. doi: 10.18632/aging.203583 34591790 PMC8544317

[36] Alshawa E, Laqqan M, Montenarh M, et al. Influence of cryopreservation on the CATSPER2 and TEKT2 expression levels and protein levels in human spermatozoa. Toxicol Rep. 2019;6:819–824. doi: 10.1016/j.toxrep.2019.08.004 31463202 PMC6706526

[37] Takiguchi H, Murayama E, Kaneko T, et al. Characterization and subcellular localization of Tektin 3 in rat spermatozoa. Mol Reprod Dev. 2011;78(8):611–620. doi: 10.1002/mrd.21352 21744413

[38] Roy A, Lin Y-N, Agno JE, et al. Tektin 3 is required for progressive sperm motility in mice. Mol Reprod Dev. 2009;76(5):453–459. doi: 10.1002/mrd.20957 18951373 PMC2657187

[39] Geng XY, Jin H-J, Xia L, et al. Tektin bundle interacting protein, TEKTIP1, functions to stabilize the tektin bundle and axoneme in mouse sperm flagella. Cell Mol Life Sci. 2024;81(1):118. doi: 10.1007/s00018-023-05081-3 38448737 PMC10917850

[40] Roy A, Lin Y-N, Agno JE, et al. Absence of tektin 4 causes asthenozoospermia and subfertility in male mice. FASEB J. 2007;21(4):1013–1025. doi: 10.1096/fj.06-7035com 17244819

[41] Jiang YZ, Yu K-D, Peng W-T, et al. Enriched variations in TEKT4 and breast cancer resistance to paclitaxel. Nat Commun. 2014;5(1):3802. doi: 10.1038/ncomms4802 24823476

[42] Wyrwoll MJ, Wabschke R, Röpke A, et al. Analysis of copy number variation in men with non-obstructive azoospermia. Andrology. 2022;10(8):1593–1604. doi: 10.1111/andr.13267 36041235 PMC9605881

[43] Aoki N, Matsui Y. Comprehensive analysis of mouse cancer/testis antigen functions in cancer cells and roles of TEKT5 in cancer cells and testicular germ cells. Mol Cell Biol. 2019;39(17):39(17. doi: 10.1128/MCB.00154-19 PMC6692122 31208979

[44] Xiong Z, Zhang H, Huang B, et al. Expression pattern of prohibitin, capping actin protein of muscle Z-line beta subunit and tektin-2 gene in Murrah buffalo sperm and its relationship with sperm motility. Asian-Australas J Anim Sci. 2018;31(11):1729–1737. doi: 10.5713/ajas.18.0025 29642674 PMC6212766

[45] Zuccarello D, Ferlin A, Garolla A, et al. A possible association of a human tektin-t gene mutation (A229V) with isolated non-syndromic asthenozoospermia: case report. Hum Reprod. 2008;23(4):996–1001. doi: 10.1093/humrep/dem400 18227105

[46] Mariappa D, Aladakatti RH, Dasari SK, et al. Inhibition of tyrosine phosphorylation of sperm flagellar proteins, outer dense fiber protein-2 and tektin-2, is associated with impaired motility during capacitation of hamster spermatozoa. Mol Reprod Dev. 2010;77(2):182–193. doi: 10.1002/mrd.21131 19953638

[47] Zhao Y, Gao N, Li X, et al. Identifying candidate genes associated with sperm morphology abnormalities using weighted single-step GWAS in a Duroc boar population. Theriogenology. 2020;141:9–15. doi: 10.1016/j.theriogenology.2019.08.031 31479777

[48] Wang Y, Huang X, Sun G, et al. Coiled-coil domain-containing 38 is required for acrosome biogenesis and fibrous sheath assembly in mice. J Genet Genomics. 2024;51(4):407–418. doi: 10.1016/j.jgg.2023.09.002 37709195

[49] Agarwal A, Sharma R, Durairajanayagam D, et al. Spermatozoa protein alterations in infertile men with bilateral varicocele. Asian J Androl. 2016;18(1):43–53. doi: 10.4103/1008-682X.153848 25999357 PMC4736356

[50] Sohail S, Tariq K, Zheng W, et al. RNAi-Mediated Knockdown of Tssk1 and Tektin1 genes impair male fertility in Bactrocera dorsalis. Insects. 2019;10(6):10(6. doi: 10.3390/insects10060164 PMC6627857 31185651

[51] Li S, Wang Q, Huang L, et al. miR-199-5p regulates spermiogenesis at the posttranscriptional level via targeting Tekt1 in allotriploid crucian carp. J Anim Sci Biotechnol. 2022;13(1):44. doi: 10.1186/s40104-022-00693-4 35418106 PMC9009052

[52] Liu S, Bian Y-C, Wang W-L, et al. Identification of hub genes associated with spermatogenesis by bioinformatics analysis. Sci Rep. 2023;13(1):18435. doi: 10.1038/s41598-023-45620-3 37891374 PMC10611713

[53] Jiang YZ, Ge L-P, Jin X, et al. Randomized phase II clinical trial and biomarker analysis of paclitaxel plus epirubicin versus vinorelbine plus epirubicin as neoadjuvant chemotherapy in locally advanced HER2-negative breast cancer with TEKT4 variations. Breast Cancer Res Treat. 2021;185(2):371–380. doi: 10.1007/s10549-020-05940-8 32975708

[54] Zheng Z, Zhou X, Cai Y, et al. TEKT4 Promotes papillary thyroid cancer cell proliferation, colony formation, and metastasis through activating PI3K/Akt Pathway. Endocr Pathol. 2018;29(4):310–316. doi: 10.1007/s12022-018-9549-0 30251060

[55] Li Y, Lin H, Shu S, et al. Integrative transcriptome analysis reveals TEKT2 and PIAS2 involvement in diabetic nephropathy. FASEB J. 2022;36(11):e22592. doi: 10.1096/fj.202200740RR 36251411

[56] Zhang R, Wu B, Liu C, et al. CCDC38 is required for sperm flagellum biogenesis and male fertility in mice. Development. 2022;149(11). doi: 10.1242/dev.200516 35587122

[57] Tsukamoto M, Hiyama E, Hirotani K, et al. Translocation of Tektin 3 to the equatorial segment of heads in bull spermatozoa exposed to dibutyryl cAMP and calyculin A. Mol Reprod Dev. 2017;84(1):30–43. doi: 10.1002/mrd.22763 27883267

[58] Ge LP, Jin X, Yang Y-S, et al. Tektin4 loss promotes triple-negative breast cancer metastasis through HDAC6-mediated tubulin deacetylation and increases sensitivity to HDAC6 inhibitor. Oncogene. 2021;40(12):2323–2334. doi: 10.1038/s41388-021-01655-2 33654196

[59] Dungan JR, Qin X, Gregory SG, et al. Sex-dimorphic gene effects on survival outcomes in people with coronary artery disease. Am Heart J Plus. 2022;17:17. doi: 10.1016/j.ahjo.2022.100152 PMC9365120 35959094

